# A Rare Case of a Distal Humerus Pathological Fracture from Klebsiella Pneumoniae Osteomyelitis: A Case Report

**DOI:** 10.5704/MOJ.2003.013

**Published:** 2020-03

**Authors:** MF Chan, EBK Kwek

**Affiliations:** 1 Department of Orthopaedic Surgery, Tan Tock Seng Hospital, Singapore; 2 Department of Orthopaedic Surgery, Woodlands Health Campus, Singapore

**Keywords:** osteomyelitis, fracture, klebsiella pneumoniae, humerus

## Abstract

Klebsiella pneumoniae is one of the leading causative organisms in pyogenic liver disease. It can cause disseminated infections, but rarely to bone, and rarely in healthy hosts. We report an unusual case of a distal humerus fracture from osteomyelitis secondary to dissemination in a non-immuno-compromised patient. The patient was surgically managed with external fixation and insertion of anti-biotic beads, in conjunction with medical therapy via culture direct antibiotics. This report highlights the diagnostic approach and treatment options for these atypical cases.

## Introduction

Klebsiella pneumoniae is a gram-negative bacterium found in the normal flora of our skin, mouth and intestine. However, it is known to cause debilitating conditions and is the leading cause of pyogenic liver disease (70.3%) in a nationwide study performed in Taiwan^1^. Disseminated infections commonly occur in immunocompromised patients – such as those with diabetes, liver or vascular disease, or a history of malignancy^2^. We present an unusual case of a patient who was treated for a left humerus pathological fracture secondary to *K.pneumoniae* dissemination from a liver abscess.

## Case Report

**Fig. 1:**
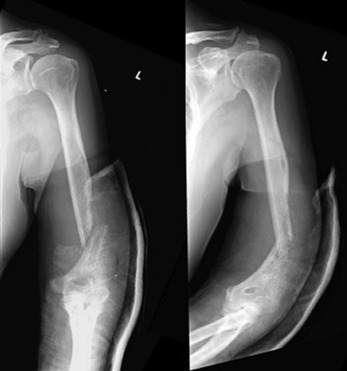
Diaphyseal fracture at the distal humerus shaft, with cortical thickening and a moth-eaten appearance suggestive of a pathological fracture.

A 72-year-old male patient with no past medical history presented with one month of left arm pain, in the absence of trauma. This was associated with unexpected weight loss of five kilograms in two weeks. Examination revealed pain over the left bicipital region, with point tenderness on the left distal humerus. Neurovascular status was intact. Blood investigations showed normal white-cell counts, raised C-reactive protein (CRP) of 210mg/L (<3 mg/L), and a raised erythrocyte sedimentation rate (ESR) of 123mm/hr (<10mm/hr). His diabetic and hepatitis screens were negative. Radiographs showed a complete diaphyseal fracture at the distal humerus shaft, with cortical thickening and moth-eaten appearance suggestive of a pathological fracture (Fig. 1).

In view of the radiographic features, tumour workup was performed; however, blood markers were normal. Computed-tomography (CT) scan of the thorax, abdomen and pelvis revealed lesions suggestive of a liver abscess, and a non-specific 0.6cm nodule in his right lung lobe. A Technetium-99m bone scan showed focal intense trace uptake at the distal humerus suspicious for a pathological fracture. Magnetic resonance imaging (MRI) of the humerus confirmed a pathological fracture of the humeral shaft with circumferential soft tissue oedema and contrast enhancement, while a multiphasic scan of the liver showed a 3.9cm drainable abscess.

**Fig. 2:**
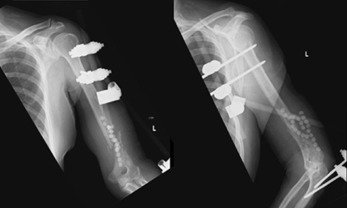
Post-operative radiographs with external fixator and antibiotic beads.

He underwent an image-guided drainage of the liver lesion and an open biopsy of the left humerus from a posterior approach. Haemo-purulent fluid was encountered upon incision. The diagnosis was confirmed when blood and fluid cultures grew *K.pneumonia*. Subsequently, he underwent interval debridement, insertion of gentamicin cement beads, and bridging external fixation from his arm to the forearm (Fig. 2). Gentamicin beads were removed one week later during a relook debridement.

As the lung nodule was sub-centimetre, decision was made for surveillance, with repeat CT-thorax in six months. The patient was managed in collaboration with infection disease physicians and treated with intravenous Cefazolin for six weeks, before conversion to oral antibiotics for three months. Subsequent blood cultures were negative for further bacterial growth, and CRP normalised after one month. ESR levels down-trended after starting therapy and normalised three months after.

**Fig. 3:**
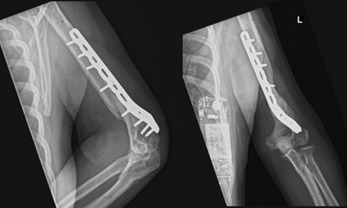
Definitive fixation with an extra-articular distal humerus locking plate, healed at three months.

The external fixator was removed at three months as inflammatory markers down-trended and the patient complained of serous discharge from the pin sites. This was converted to an extra-articular distal humerus locking plate applied in compression with bone graft to the fracture site (Fig. 3). Tissue and fluid cultures taken intra-operatively were negative for bacterial growth.

## Discussion

This is a rare case of disseminated *K.pneumoniae* infection resulting in osteomyelitis and pathological fracture, in an immune-competent host with no past medical history, recent hospital admissions or high-risk exposure.

In Keller *et al*^1^, population study of over 70 000 *K.pneumoniae* liver abscesses, 28% had presence of a metastatic foci to the lung, heart, genitourinary system, and eye, to name a few. Dissemination causing osteomyelitis was reported only as 0.6%.

Hence, hematogenous spread of infection to bone is rare, discussed only in scattered case reports and paediatric cases^3^. However, in recent years, new hypervirulent Kp strains (especially serotypes K1 and K2) have been associated with life-threatening infections (bacteraemia, liver abscess, and meningitis) and distant metastases in younger immune-competent hosts. Increased virulence is related to the presence of the K-antigen, producing a hyper-viscous capsule leading to growth of mucoid colonies on culture plates that resists phagocytosis^4^.

Cases of *K.pneumonia* osteomyelitis resulting in pathological fractures in adults are also far and few between. Previous case reports published of osteomyelitis related pathological fractures show varying treatments such as surgical debridement with splinting for immobilisation, versus surgical debridement with skeletal traction acutely and interval internal fixation thereafter. Ideally, direct internal fixation should be avoided in a contaminated environment due to risks of biofilm formation resulting in resistance to antibiotic therapy, and high rates of non-union^5^. Ultimately, there is insufficient evidence in the literature to identify a gold standard of treatment for such presentations.

Our approach to the case was to first consider and rule out a metastatic focus in a patient with osteomyelitis. Testing of blood aerobic and anaerobic cultures before antibiotic treatment is an essential step to guide treatment, should infection be suspected. Blood investigations may be non-specific, and hence a thorough history with systems review should be performed. As *K.pneumoniae* is the most common isolate from pyogenic liver disease, further workup is required should there be evidence in blood or tissue cultures. This includes specific investigations such as liver function testing and dedicated imaging such as ultrasonography, CT or MRI scans. Multi-disciplinary input from physicians for management of septicaemia with supportive measures and culture directed antibiotics is recommended.

We modelled our surgical management of the pathological fracture with that of infected non-unions. Rather than using intramedullary antibiotic-impregnated cement rods, we opted for antibiotic beads and application of an external fixator. This allowed us to achieve preliminary stabilisation while controlling and obliterating the infection. It served well as a temporising measure until the infection was fully eradicated and could be replaced with a definitive fixation option such as the distal humerus plate, which would otherwise be unsuitable in an acutely septic environment.

In conclusion, pathological fractures from hematogenous spread of an organism are rare. While it occurs more often in the immunocompromised population, any patient presenting with a pathological fracture has to be worked up adequately to first rule out malignancy, followed by a broad assessment to identify a source of hematogenous spread. Treatment requires a multi-disciplinary approach with physicians on board for management of sepsis, and the surgical team for definitive therapy.
